# Deeper Insight of the Conformational Ensemble of Intrinsically Disordered Proteins

**DOI:** 10.1021/acs.jcim.4c00941

**Published:** 2024-07-26

**Authors:** Oskar Svensson, Michael J. Bakker, Marie Skepö

**Affiliations:** † Division of Computational Chemistry, Department of Chemistry, Lund University, P.O. Box 124, SE-221 00 Lund, Sweden; ‡ NanoLund, Lund University, Box 118, 22100 Lund, Sweden; § Faculty of Pharmacy in Hradec Králové, Charles University, Akademika Heyrovského 1203/8, 500 05 Hradec Králové, Czech Republic

## Abstract

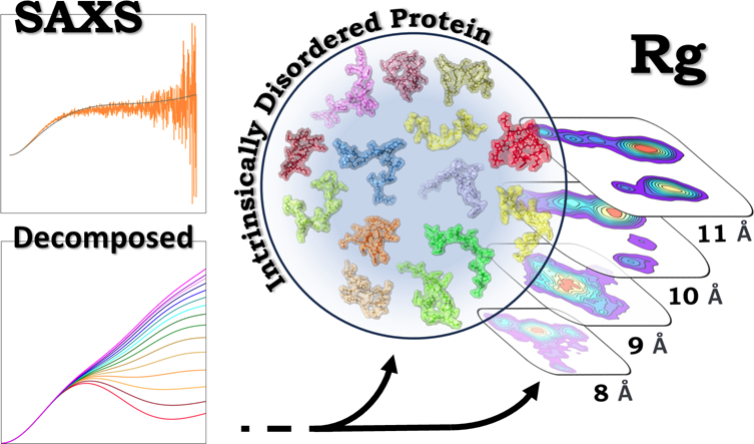

It is generally known that, unlike structured proteins, intrinsically disordered proteins, IDPs, exhibit various structures and conformers, the so-called conformational ensemble, CoE. This study aims to better understand the conformers that make up the IDP ensemble by decomposing the CoE into groups separated by their radius of gyration, *R*_g_. A common approach to studying CoE for IDPs is to use low-resolution techniques, such as small-angle scattering, and combine those with computer simulations on different length scales. Herein, the well-studied antimicrobial saliva protein histatin 5 was utilized as a model peptide for an IDP; the average intensity curves were obtained from small-angle X-ray scattering; and compared with fully atomistic, explicit water, molecular dynamics simulations; then, the intensity curve was decomposed with respect to the different *R*_g_ values; and their secondary structure propensities were investigated. We foresee that this approach can provide important information on the CoE and the individual conformers within; in that case, it will serve as an additional tool for understanding the IDP structure–function relationship on a more detailed level.

## Introduction

In contrast to globular proteins, intrinsically disordered proteins, IDPs, adopt many different conformations in solution.^1,2^ This transient structural nature means that IDPs are well suited to adopt various functions; however, it also makes them challenging to study using many traditional biophysical techniques.^3−5^ IDPs are better described by a conformational ensemble, herein termed CoE, of several structural states, the so-called conformers, between which the protein can interchange. The CoE of IDPs represents a dynamic and versatile paradigm in protein science. The flexibility and heterogeneity of the ensemble underpin the IDPs’ functional promiscuity and adaptability, and the greater familiarity leads to an understanding that they are key players in many complex biological processes. Thus, understanding the CoE of IDPs is an important step in unraveling the mechanisms of IDP function and their roles in health and disease.

Solution techniques, such as small-angle X-ray scattering (SAXS) and small-angle neutron scattering (SANS), play a central role in structural studies of IDPs and can be used to characterize IDPs in solution at a broad range of concentrations^6,7^ to yield information on the structural properties of the protein, the oligomerization state, and the protein–protein interactions within the solution.^8−11^ Because of the low resolution, it is generally necessary to combine SAXS with additional experimental sources and use molecular simulations to interpret and integrate the data.^12,13^ Herein, a more in-depth analysis of the CoE of IDPs is implemented by decomposing the CoE to better understand the distinct structural states that IDPs populate. Decomposition involves identifying and characterizing the various substates within the ensemble and the transitions between them. In this study, the CoE is decomposed by dividing it into several groups with respect to the radius of gyration, *R*_g_. This manuscript demonstrates the approach for the intrinsically disordered antimicrobial saliva protein histatin 5, Hst5, and focuses on the variations in the scattering features of CoEs, applies dimensionality reductions/clustering algorithms, and investigates their secondary structure propensities.

## Methodology

### Molecular Dynamics

Atomistic molecular dynamics, MD, simulations were performed with the GROMACS package version 2021.^14,15^ Simulations consisted of a single Hst5 chain in water and 150 mM NaCl to mimic the physiological conditions. The force field, AMBER99SB-ILDN, with the water model TIP4P-D was applied for all simulations.^16,17^ The starting configuration of Hst5 was a linear chain and was constructed with Avogadro version 1.2.0.^18^ Both termini were set to be charged, the side chains were modeled at neutral pH, and the histidine residues were neutral. This gave Hst5 a net charge of +5e. A dodecahedron box was used with standard periodic boundary conditions applied in all directions. The peptide was inserted into the box so that all atoms were at least 1 nm from the edges. Solvent molecules were replaced by ions, NaCl, to attain the appropriate salt concentration and keep the system neutral. The primary structure of Hst5 is given below where red signifies positively charged residues, blue signifies negatively charged residues, and green signifies histidine residues.
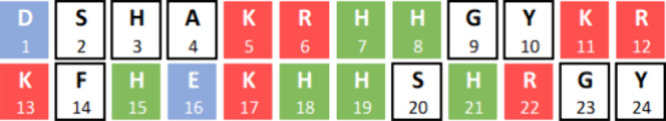
GROMACS’ leapfrog integration algorithm was used for the equations of motion with a 2.0 fs time step. A Verlet cutoff scheme was applied for nonbonded and short-ranged interactions with a cutoff of 12 Å. Dispersion corrections were applied to the energy and pressure. The particle mesh Ewald, PME, method^19^ was used for long-ranged electrostatics with cubic interpolation and grid spacing of 1.6 Å. Bonds containing hydrogen were constrained using the LINCS algorithm.^20^ The temperature was set to 298 K by applying the Noose–Hover thermostat^21^ with a temperature fluctuation time of 1.0 ps. A separate coupling group was used for the peptide. The pressure was set isotropically by applying the Parrinello–Rahman barostat.^22^ Pressure coupling used a time constant of 5.0 ps and compressibility of 4.5 × 10^–5^ bar^–1^. Energy minimization used the steepest descent algorithm. Equilibration was carried out in three steps: (i) 0.5 ns in the *NVT* ensemble, (ii) 0.5 ns in the *NPT* ensemble, and (iii) 1.0 ns in the *NPT* ensemble, where *N*, *P*, *V*, and *T* correspond to a constant number of particles, pressure, volume, and temperature, respectively. Position restraints were used for the peptide during all equilibration steps. Five trials were simulated with a production run of 2 μs each, for a total of 10 μs.

### Trajectory Analysis

**Figure 1:**
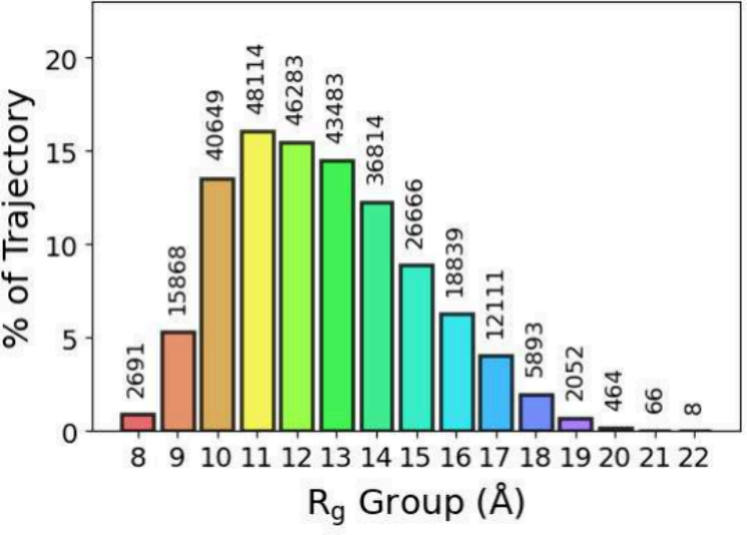
Relative distribution of the decomposed conformational ensemble, CoE, upon decomposition into different radius of gyration, *R*_g_, groups. The actual number of frames is annotated above each bar.

The first 100 ns of each simulation trial were discounted to avoid influence from the initial folding of the straight chain. All the trial trajectories were then concatenated using GROMACS’ gmx *trjcat*.^14,15^ From this, a concatenated trajectory of 300 000 frames was collected. Using an in-house Python script and the GROMACS’ gmx *gyrate* command,^14,15^ the frames were sorted on the basis of *R*_g_. This leads to the formation of *R*_g_ groups with differing numbers of frames (see Figure 1). The Python script produces mimics of GROMACS frame index files, which can be used to create individual trajectory files for each *R*_g_ group using GROMACS’ gmx *trjconv* command;^14,15^ these files can then be used for analysis.

Pair distances, Ramachandran plots, and hydrogen bonding were determined using GROMACS’ *pairdist*, *rama*, and *hbond*, respectively. Ramachandran’s data was plotted as a 2D histogram, which allowed integration in determining the fraction of the total data points inhabiting the bins within a certain plot region.^14,15^ Clustering was performed using gmx *cluster* with the GROMOS^23^ method with a 0.7 nm cutoff. Scattering profiles were calculated for each of the 300 000 frames using CRYSOL version 3.2.1 with a contrast of hydration shell value set to 0; all other parameters used default settings.^24^ Using the previously mentioned in-house script, an average scattering profile for each detected *R*_g_ group and an average profile for all frames used were created. Figure 1 shows all of the found *R*_g_ groups and the number of frames within each. The intraparticle distance distribution function, *P*(*r*), was determined for each average using PRIMUS version 3.2.1.^25^ Deeptime^26^ was used to implement time-lagged independent component analysis, tICA. This approach enabled us to construct free energy and solvent-accessible surface area, SASA, landscapes with aid from the Pyemma^27^ and MDtraj^28^ packages, respectively, as described in previous works.^29,30^ χ^2^ was calculated to compare experiment data to simulation data using eq 1 where *N* is the number of data points, *E*_*i*_ is the experimental data point, and *S*_*i*_ is the simulated data point.^31^
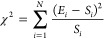
1

## Results and Discussion

### Average Histatin 5 Behavior

**Figure 2:**
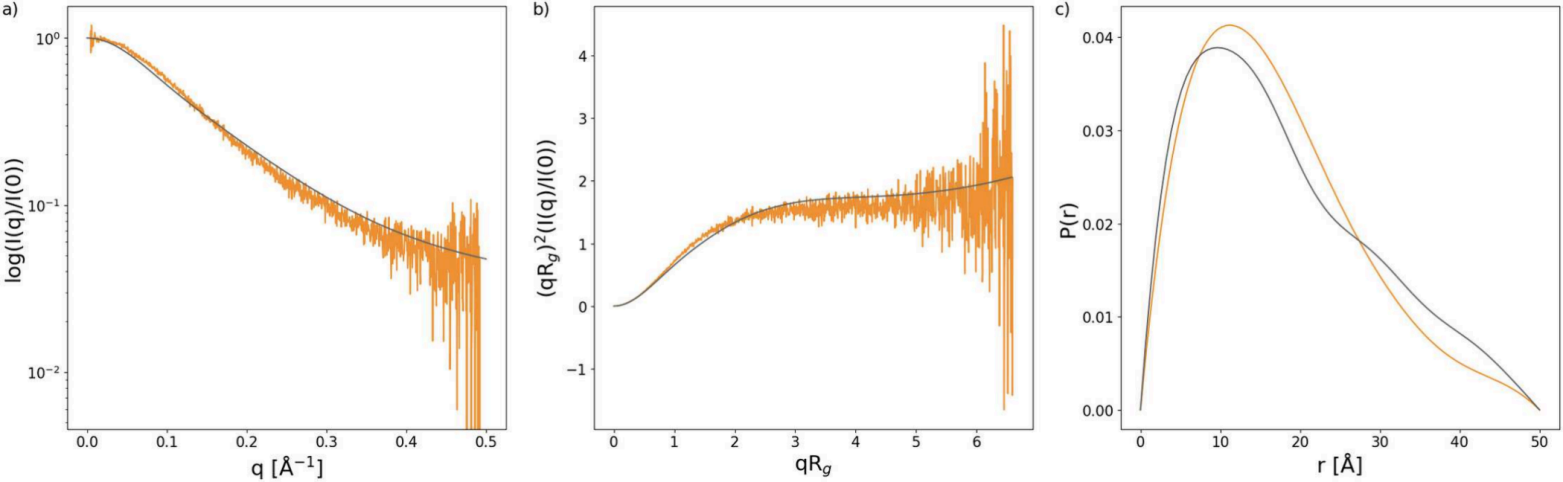
(a) The scattering intensity, *I*(*q*), vs the scattering vector *q*, (b) the Kratky plot, and (c) the intraparticle distribution function, *P*(*r*), of histatin 5. The orange curve corresponds to experimental data,^36^ and the black curve corresponds to molecular dynamics simulations. The AMBER99SB-ILDN force field and TIP4P-D water model were applied for the latter. The SAXS data was obtained for a 3.12 mg/mL concentration at an ionic strength of 150 M and pH 7. The temperature was set to 25 °C.

The average behavior of Hst5 using MD simulations and SAXS has been investigated in several studies.^12,32−35^Figure 2 shows the typical features of SAXS compared to MD for three different analyses: (a) *I*(*q*) versus the scattering vector, *q*, (b) the Kratky plot, and (c) the *P*(*r*) with χ^2^ values of 0.72 and 3.27 for the two former. In general, there is good agreement between the two approaches. It is also shown that the average structure is a random coil, which is expected from an IDP and earlier published results.^32,36^

### Decomposition of the Conformational Ensemble

#### Small-Angle X-ray Scattering

**Figure 3:**
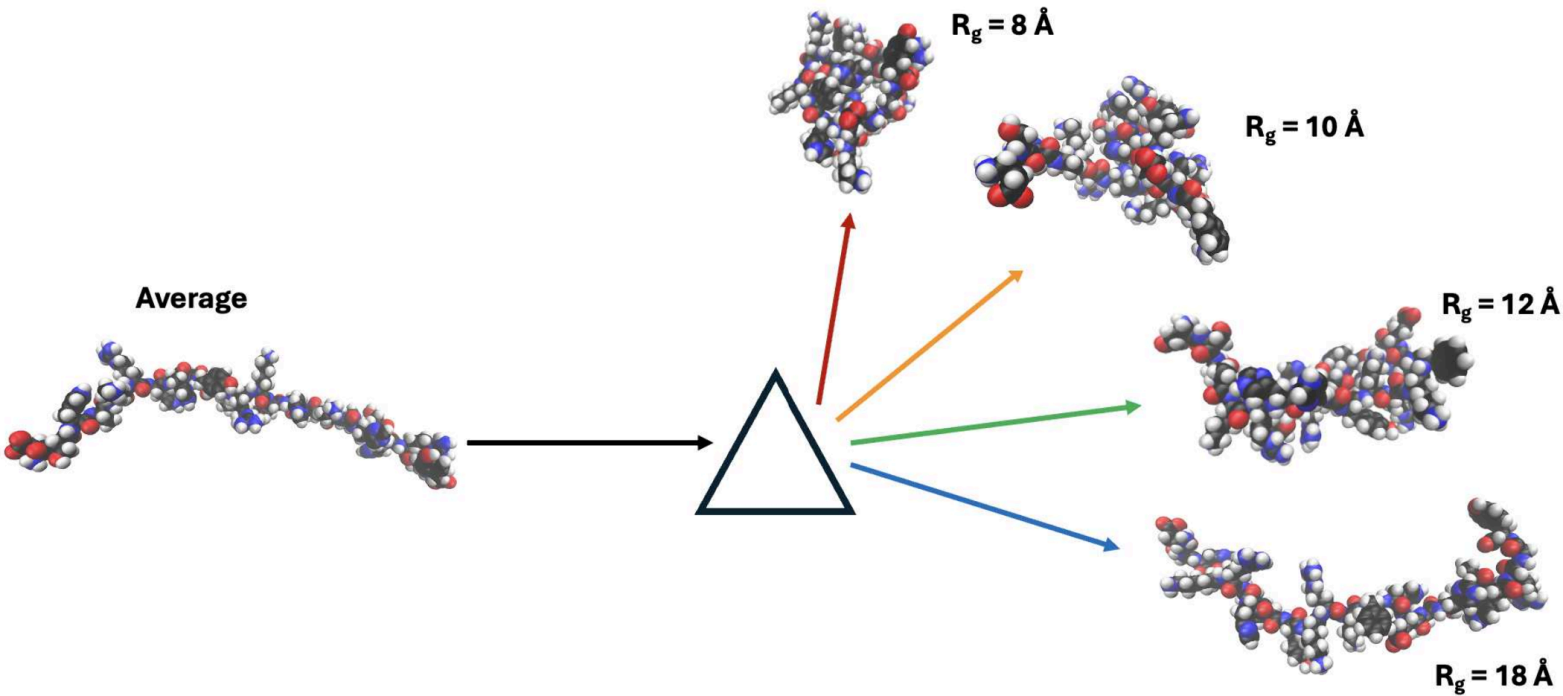
A visual example of decomposition of the conformational ensemble, CoE. Here, the average is decomposed into four groups with a radius of gyration, *R*_g_, of 8, 10, 12, and 18 Å, respectively.

As mentioned above, from SAXS, the average structure of an Hst5 peptide can be achieved from CoE, and by combining it with MD simulations, we can obtain more detailed information. As shown in Figure 1, *R*_g_ has a wide distribution from 8 to 22 Å and is relatively smooth with an average *R*_g_ of approximately 13 Å, which correlates well with the experimental values.^36^ Similar to how a prism disperses white light into its constituent wavelength, a decomposition analysis contextualizes the constituent conformation types, which comprise the average seen in Figure 2; Figure 3 shows a visualization with snapshots.

**Figure 4:**
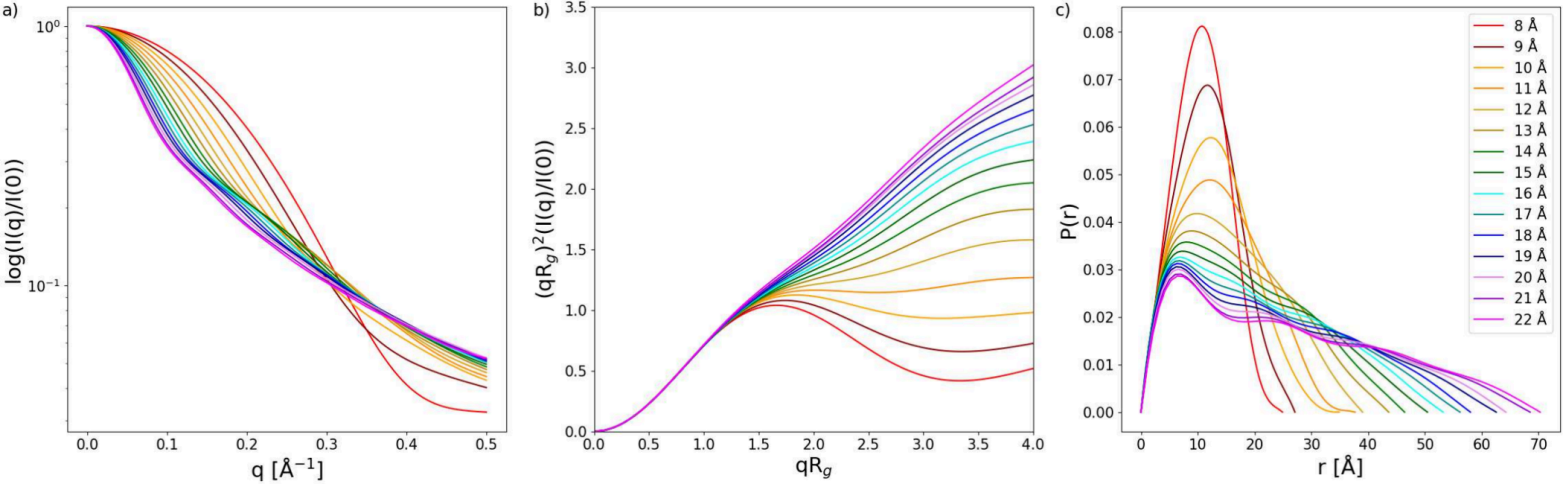
(a) The scattering intensity, *I*(*q*), versus the scattering vector *q*, (b) the Kratky plot, and (c) the intraparticle distribution function, *P*(*r*), of histatin 5 obtained from molecular dynamics simulations from each representative radius of gyration, *R*_g_, group in Figure 1. The color code is shown with the number corresponding to the given *R*_g_.

From the data shown in Figure 1, it is demonstrable that there is a great variety of structures and conformations in the CoE. This is verified in Figure 4 where the decomposed SAXS data is shown. For example, Figure 4a shows that there is a great variance in *R*_g_, as already shown in Figure 1; the Kratky plot in Figure 4b shows that the shape is clearly different from a rigid rod, via a random coil, to a semiglobular/globular state for *R*_g_ group = 8 Å. The *P*(*r*) similarly shows a decreasing maximum distance within the peptide, *r*_max_, with decreasing *R*_g_. Monomodality in the distance distribution increases with decreasing *R*_g_ with a slight indication of double peak patterns.

#### Protein–Protein/Protein–Solvent Interactions

**Figure 5:**
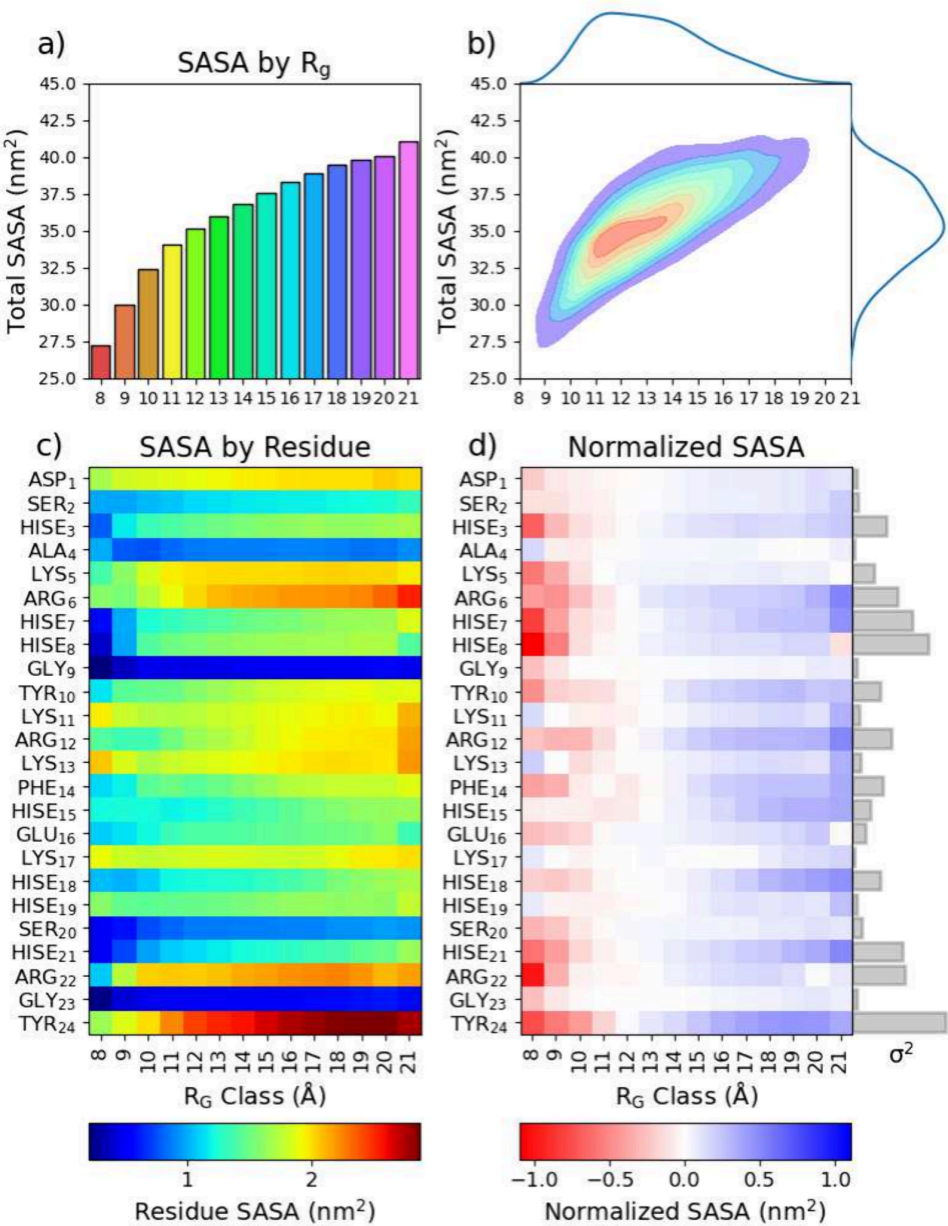
Solvent-accessible surface area, SASA, variations as a function of the radius of gyration, *R*_g_, groups represented as a (a) bar chart and (b) 2D KDE plot, as well as heatmaps of SASA by *R*_g_ class per residue, both (c) non-normalized and (d) normalized, as well as the variance detected by residue as a bar chart in gray.

From SASA, a positive correlation between SASA and *R*_g_ is noticed, as seen in Figure 5a, since as the protein size increases its surface area also increases, which implies a higher SASA value. As the protein becomes more compact, the SASA value decreases. Therefore, proteins with higher *R*_g_ values tend to have higher SASA values than those with lower *R*_g_ values. Figure 5b shows the kernel density estimation (KDE) distributions of the conformations as a function of *R*_g_ and the total SASA, thereby demonstrating that at high and low *R*_g_ there is a significantly lower variation in the expressed solvent area than more median conformations. This represents that at median range *R*_g_ states the amount of accessible surface area is far more flexible.

This trend becomes more interesting when analyzing the decomposed *R*_g_ groups, highlighting that exposed areas from different residues are differently impacted by *R*_g_. Here, SASA was directly investigated on a residue-by-residue basis (see Figure 5c). However, comparing residues becomes challenging as the size and shape of different residues fluctuate depending on the residue being investigated. To compensate, a normalized SASA^37^ was determined using the “expected” solvent values as a baseline for each residue, and then, the difference was computed between the expected and the observed, as seen in Figure 5d. By separating the CoE into *R*_g_ groups and residues and normalizing against the expected surface area, a technique emerges to investigate not only the parts of the protein that are most exposed but also reveal the residues’ likelihood to change upon expansion or contraction and their subsequent dependence on *R*_g_. Most noticeably, this variance is highly pronounced in charged or polar residues, such as Y24, R6-H8, H21, or R22. Among the histidine residues, some residues are particularly prone to changes in the *R*_g_, such as H8, while others are negligibly influenced, such as H19.

**Figure 6:**
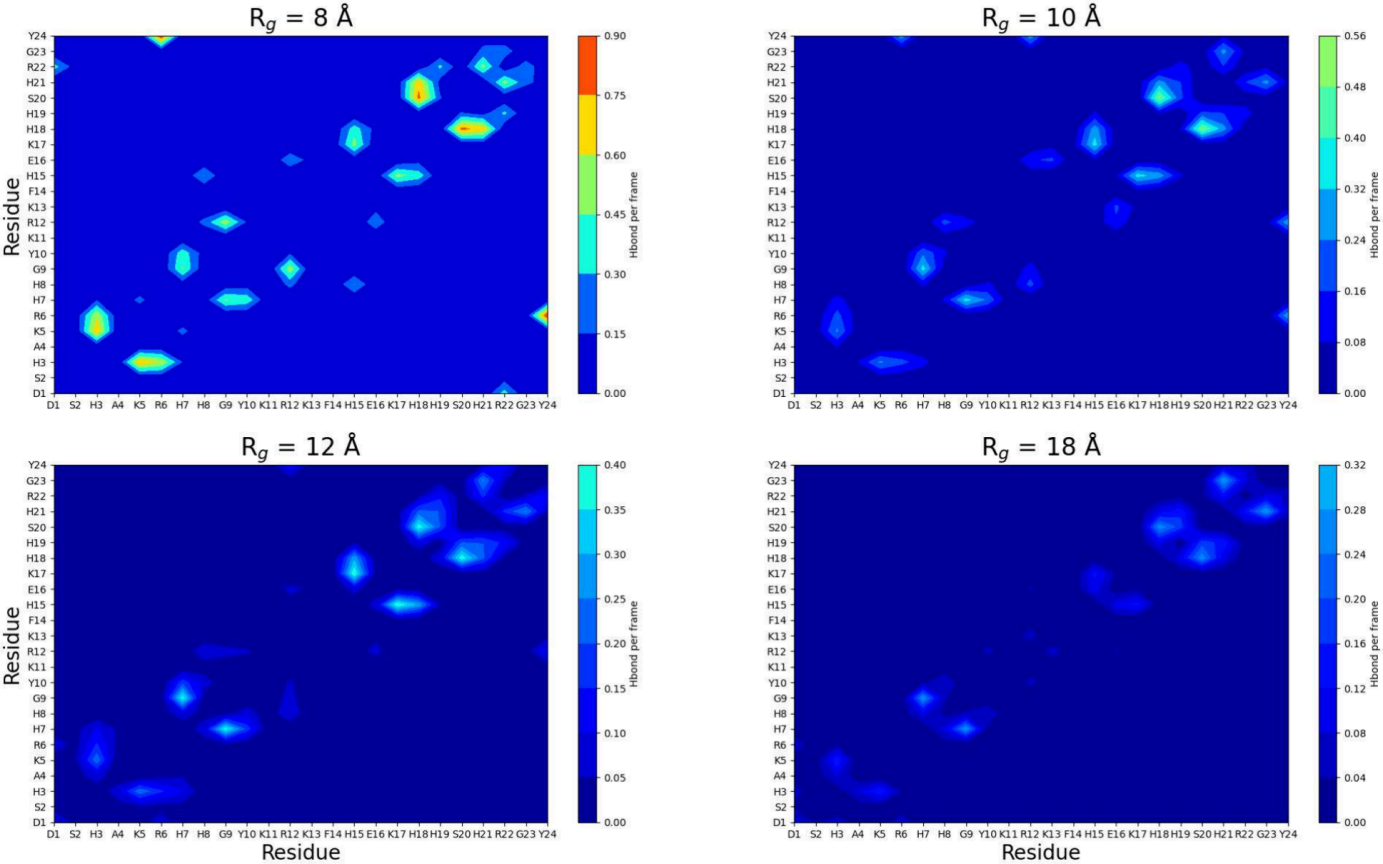
Hydrogen bond contact maps for all selected radius of gyration, *R*_g_, groups.

The variations in SASA observed are possibly due to transient intramolecular interactions within the chain. Thus, the changes in hydrogen bonding within four selected *R*_g_ groups were tracked (see Figure 6). The pattern is consistent; however, the intensity increases with an increased compactness. Table 1 summarizes all of the major residue pairs involved in hydrogen bonding. It is observed that several residues with high variance of SASA partake in key interactions, which vary with compactness, such as Y24 and R22, although there are also cases, such as H19, with minor SASA variance. Overall, it does not appear to be a correlation between SASA variance and hydrogen bonding; however, compactness leads to a smaller distance between residues, which may facilitate hydrogen bonding; this explains why hydrogen bonding increases at lower compactness.

**Table 1:** A List of All Major Hydrogen Bonding Pairs That Can Occur within Histatin 5 with the Residues in the Primary Sequence Given in a One-Letter Code

residue X	H3	H3	R6	H7	H7	G9	H15	H5	H18	H18	H19	H21
residue Y	K5	R6	Y24	G9	Y10	R12	K17	H18	S20	H21	R22	R22

**Figure 7:**
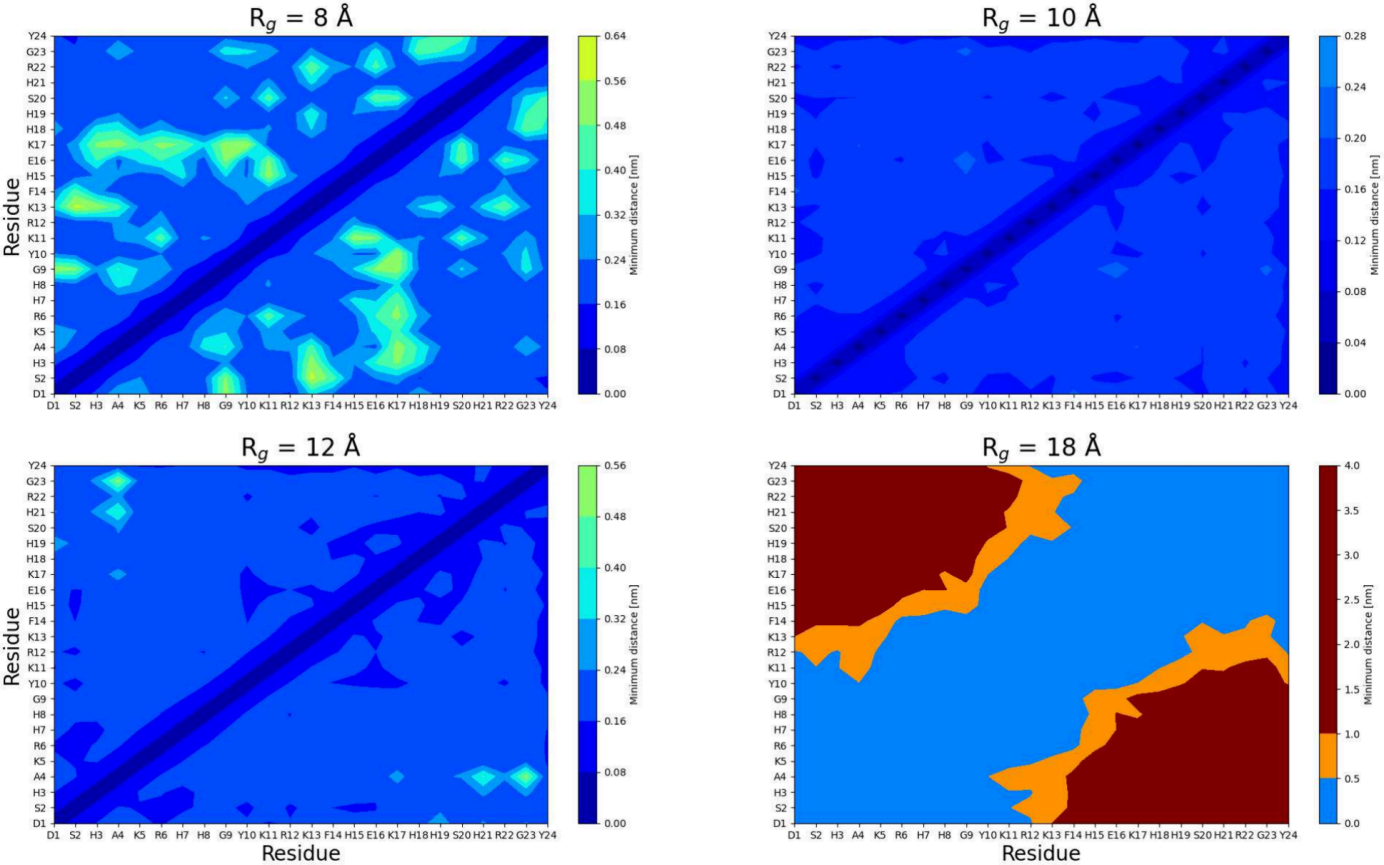
Contact maps for the full trajectory and the selected groups of radius of gyration, *R*_g_.

The intramolecular interactions were further examined using contact maps (see Figure 7). The group of *R*_g_ 18 Å attains the pattern of a rodlike shape, which is expected considering the result in Figure 4b. Conversely, the groups of *R*_g_ 10 Å and *R*_g_ 12 Å are quite similar except for the larger distances observed of residues H21 and G23, likely due to the decrease in compactness, whereas the group of *R*_g_ 8 Å, interestingly, displays larger distances when compared with the groups of *R*_g_ 10 Å and *R*_g_ 12 Å despite being considerably more compact. When compared to the SASA results, G9, K13, and K17 all attain relatively high SASA values for the *R*_g_ group, which display large distances to the terminal sections of the chains, which show low SASA values for the group of 8 Å. This could indicate partially folded conformations where certain residues are situated in more solvent-accessible positions due to intramolecular interactions. It is notable that K13 and K17 are not in Table 1, although G9 is. This interpretation is supported by Figure 4b.

#### Protein Fluctuation and Landscape

**Figure 8:**
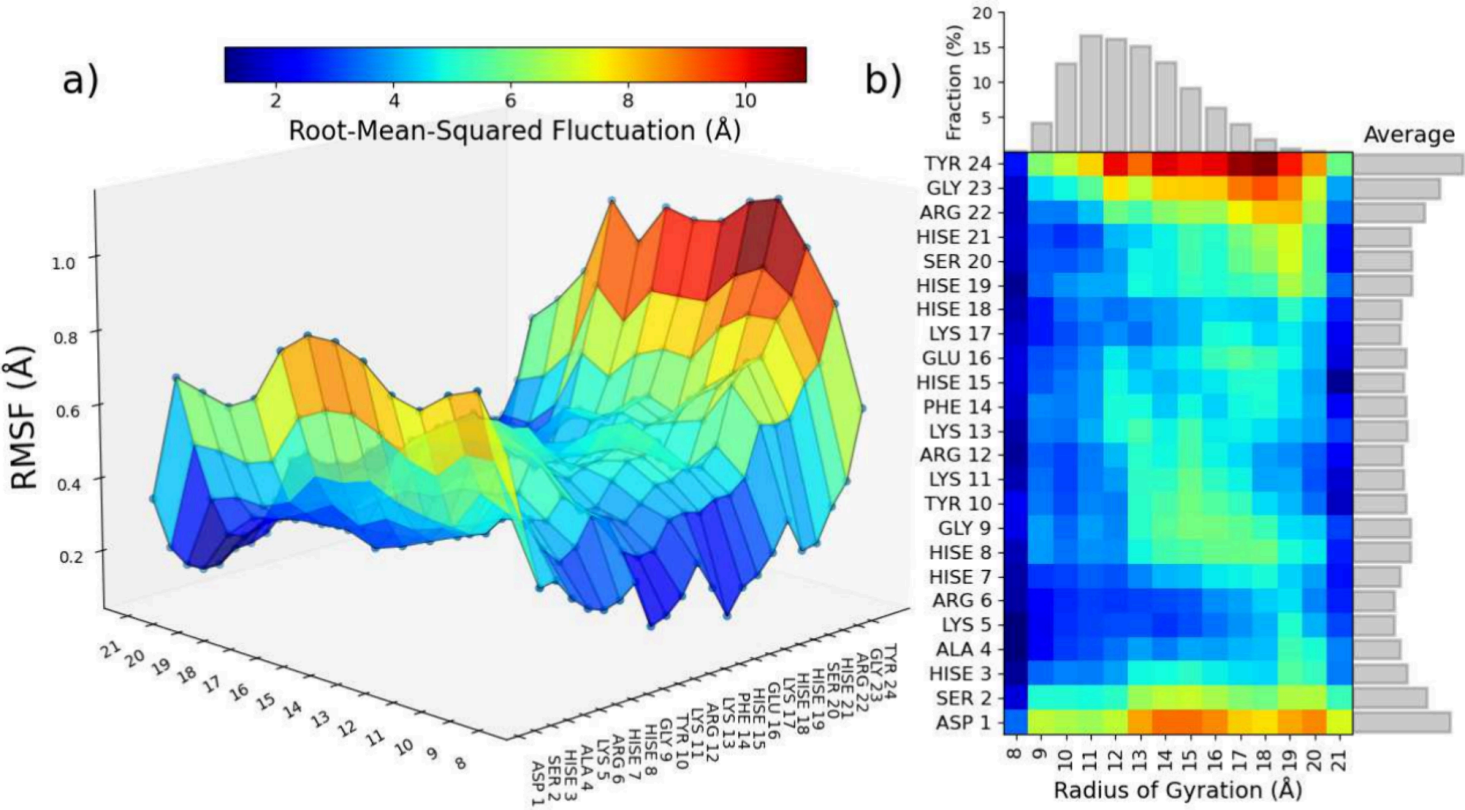
Root-mean-squared fluctuation, RMSF, determined for each radius of gyration, *R*_g_, group as a (a) 3D surface plot and (b) heatmap with the fraction of each group and the average RMSF plotted for reference.

The expected behavior of Hst5 follows similar behavior for IDPs, as seen in the average RMSF in Figure 8. The tails have far higher fluctuations than internal residues, which is explainable because they are significantly more exposed than the internal regions, indicating that the internal areas possess higher stability and structure than the tails. Notably, the C-terminal, T24, fluctuates the most with the lowest being around K5. Implementing *R*_g_ decomposition, one can uncover patterns in the behavior not previously exposed. For example, several residues, H8–R12, exhibit greatly enhanced fluctuation but only at *R*_g_ between 11 and 16 Å. Another highly flexible region includes F14–K17, although this behavior is only revealed in *R*_g_ groups between 10 and 13 Å. The RMSF and *R*_g_ decomposition analysis provides complementary information about the flexibility and stability of Hst5: while the RMSF shows the overall fluctuations of each residue, the *R*_g_ decomposition allows us to observe how the flexibility changes as a function of the size of the protein.

**Figure 9:**
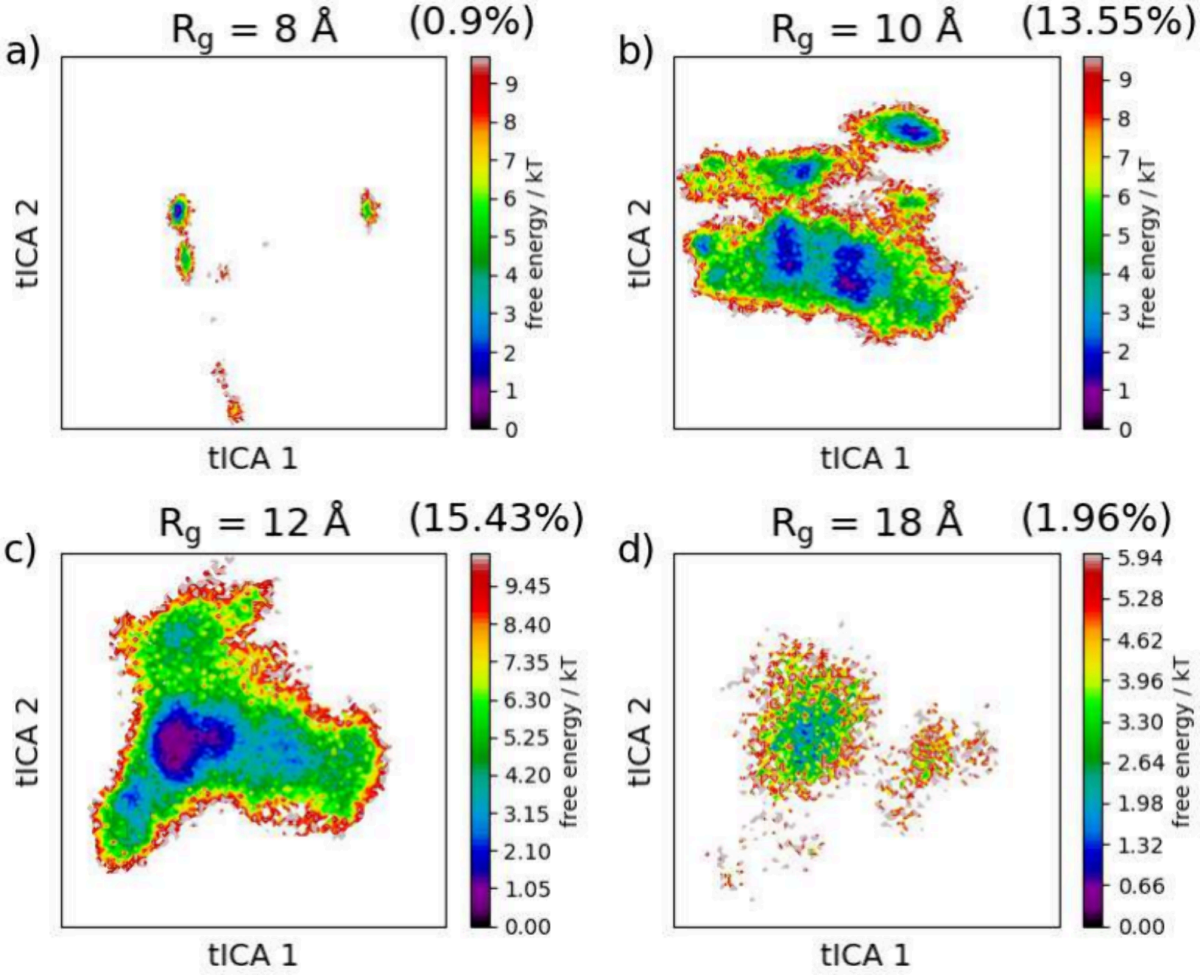
Time-lagged independent component analysis, tICA; free energy landscapes for the four selected groups of radius of gyration, *R*_g_; and the percentages of the complete set of conformations that each group encompasses. Free energy is expressed in kT at 298 K.

Within the decomposed data, fluctuations may also occur within the free energy landscape. This is because, unlike when creating an average landscape, characteristics from less prevalent *R*_g_ groups are not hidden. Figure 9 shows free energy landscapes on the basis of a tICA analysis of four selected groups of *R*_g_. The landscapes vary drastically, which indicates large differences between *R*_g_. Most notable is the presence of multiple free energy minima in group 10 Å, which are not seen in group 12 Å. These minima indicate a larger variation of conformation at group 10 Å than within group 12 Å since there are several equally stable regions rather than a singular well. The tICA analysis coupled with the protein fluctuations also further contextualizes the Hst5 IDP nature since higher fractions of the complete set of conformations tend to align with higher levels of protein fluctuations. This also has implications for conformational entropy: the more extreme *R*_g_ groups are restrictive since, in this instance, they necessitate rodlike or partially globular structure (see Figure 4). Based on the data in this section, Hst5 tends toward higher conformational entropy and less restrictive conformations, which is evidence of a random coil, as seen in Figure 2.

**Figure 10:**
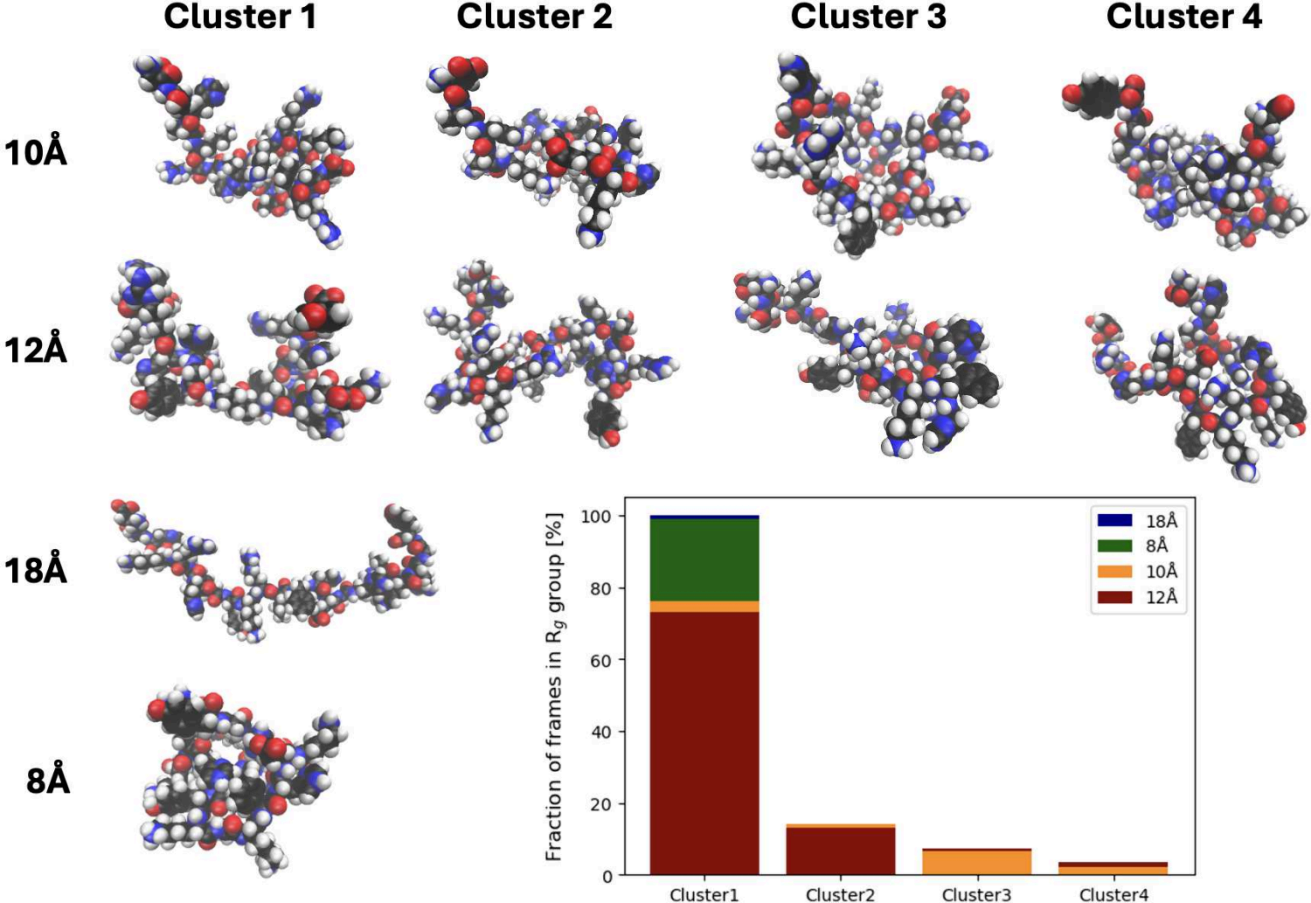
Snapshots representing the average structure of each significant cluster attainable from the cluster analysis. A visualization of frame distribution throughout the clusters is also presented.

The structural variation indicated in Figure 9 was further investigated through clustering structures (see Figure 10). Applying an identical method for all chosen *R*_g_ groups, it is observed that 8 and 18 Å attain a single significant cluster, while 10 and 12 Å show four each. The average structures in Figure 10 vary in compactness between *R*_g_ groups, as expected. Within 10 and 12 Å, there are minor changes within each cluster. This implies fluctuations within the intramolecular interactions. The 10 and 12 Å groups have similar distributions between the clusters, which do not elaborate on the results in Figure 9.

#### Secondary Structure Analysis

**Figure 11:**
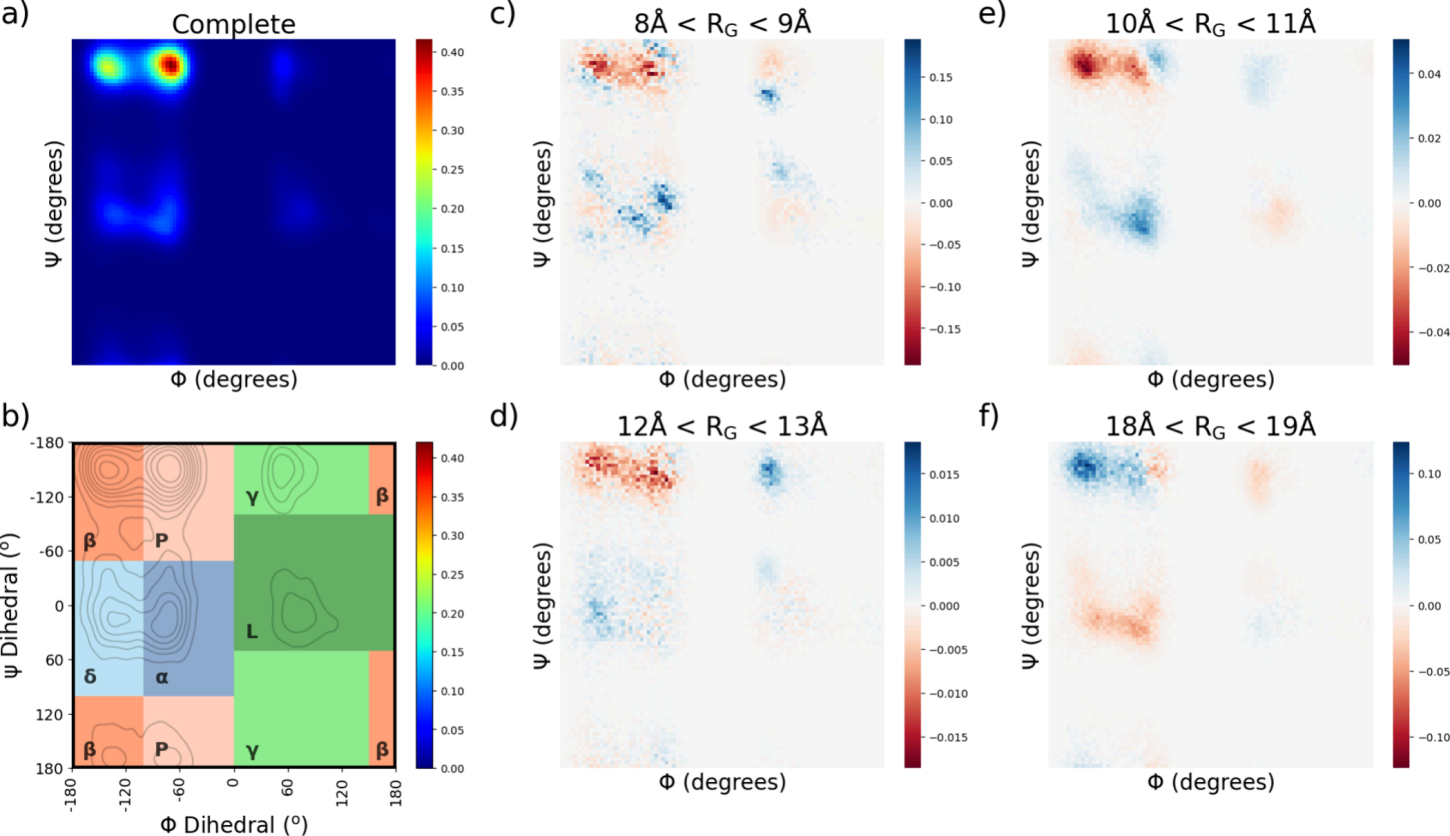
Integrated Ramachandran plots displaying (a) the complete trajectory, (b) important secondary structural regions, and the difference in dihedral preference for the groups of (c) 8, (d), 10, (e) 12, and (f) 18 Å.

Ramachandran plots were determined from the trajectories to examine the secondary structure further. The different regions within the plots correspond to a variation of secondary elements, which can be estimated on the basis of the occurrence of angle pairs. Figure 11 shows Ramachandran plots for the complete and decomposed data. Four distinct regions were selected on the basis of the distribution of the angle pairs found throughout the simulation, and there is an apparent variation within the regions on the basis of the compactness of the chain.

**Figure 12:**
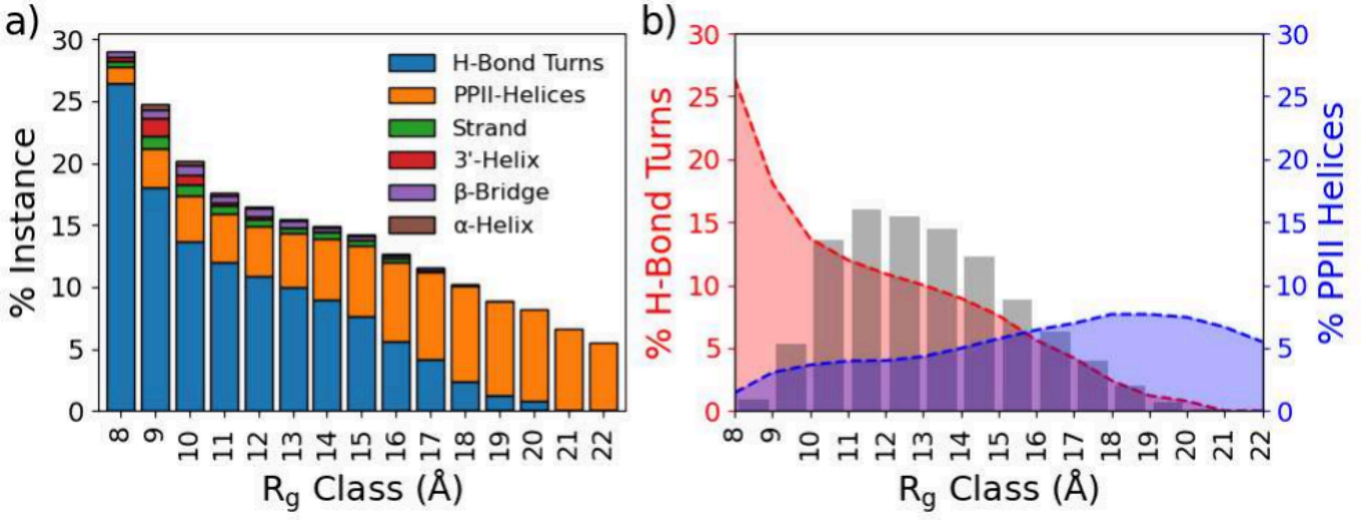
Secondary structure averaged from the different radius of gyration, *R*_g_, groups plotted as (a) a stacked bar chart and (b) shaded line plots with the *R*_g_ distribution shaded for reference.

The significant deviations noticed in *R*_g_ group 8 Å might be due to transient secondary structure. The appearance of the secondary structure was investigated using the DSSP algorithm (see Figure 12). The algorithm does detect elements of secondary structure in several of the *R*_g_ groups. All groups display PPII-helix elements, which Hst5 is known to adopt;^33^ however, *R*_g_ groups 8, 10, and 11 Å show some presence of other structural components as well. The overall level of elements remains low in all groups except hydrogen-bonded turns. There is a clear trend of increasing hydrogen-bonded turns with lowering *R*_g_, and the reversed trend is observed for PPII-helices (Figure 12b).

The regions were integrated, and Table 2 shows the integral fraction for each secondary structure element for each selected *R*_g_ group and the nondecomposed data; this fraction acts as occurrence estimation. There is a significant presence of β-sheet, PPII-helix, and α-helix, as well as a lesser presence of the left-handed (LH) helix. α-Helical elements seem to increase with increased compactness, and the β-sheet appears to decrease.

**Table 2:** Regions of the Ramachandran Plots Displayed as Phi Range, Psi Range, Secondary Structure Element Associated with the Region, Integral Fraction in Percent for the Selected *R*_g_ Groups, and the Average of the Full Trajectory

	β-sheet	PPII helix	α-helix	LH helix
ϕ	[−170, −120]	[−90, −40]	[−110, −40]	[30, 70]
ψ	[120, 180]	[100, 180]	[−50, 10]	[10, 50]
group	region 1	region 2	region 3	region 4
8 Å	19	26	24	4
10 Å	22	30	19	3
12 Å	24	30	17	3
18 Å	30	33	10	2
average	4	3	3	2

## Conclusions

Here, we have demonstrated an approach to decompose the CoE of IDPs with respect to *R*_g_ groups. The MD data of the average behavior and full trajectory are compared with experimental SAXS data and depict good agreement. The results show that CoE consists of a wide variety of different structures that, as such, are hidden in the low-resolution SAXS data. While this specific method for decomposition of CoE with respect to the *R*_g_ was demonstrated with positive results, it can be further developed and adapted to suit the specific study’s purpose and goals. As an initial study, we focused on the single chain. There are, of course, several areas that need to be further studied and validated. For example, certain *R*_g_ groups, such as 8 Å, suffer from lower levels of sampling. Attempts should be made to alleviate this concern potentially through enhanced sampling methods. The presented procedure is also for a noninteracting ideal system, and there is a need to further develop the method to suit more complex systems, such as longer IDPs with a more complex primary sequence, as well as self-associating systems.

## Data Availability

All MD simulation trajectories are available upon request. All input files to run the simulations, the scripts implemented for the CoE decomposition, and our current implementation of the machine learning models can be downloaded from the GitHub repository: https://github.com/skepo/A-Deeper-Insight-of-the-Conformational-Ensemble-of-Intrinsically-Disordered-Proteins.git.

## Supporting Information Available

Various visual representations and correlations of the total SASA and *R*_g_ in the system, including box plots, a time-dependent representation, and correlations with three specific residues, and the *R*_g_ vs total SASA plots, individual and integrated Ramachandran plots color-coded by *R*_g_, and correlations between total SASA and *R*_g_ per residue (PDF)
