# Supplementary material for: Deeper Insight of the Conformational Ensemble of Intrinsically Disordered Proteins
Source: J Chem Inf Model. 2024 Jul 26;64(15):6105–14. doi: 10.1021/acs.jcim.4c00941 (PMC11323008; doi:10.1021/acs.jcim.4c00941)
Supplement: Supplementary file 1 — ci4c00941_si_001.pdf [file ci4c00941_si_001.pdf]

Supporting Information for A Deeper Insight of the Conformational Ensemble of Intrinsically Disordered Proteins

Oskar Svensson,[†,‡] Michael J. Bakker,[¶] and Marie Skepö[*,†,‡]

†*Division of Computational Chemistry, Department of Chemistry, Lund University, P.O. Box 124, SE-221 00, Lund, Sweden*

‡*NanoLund, Lund University, Box 118, 22100 Lund, Sweden*

¶*Faculty of Pharmacy in Hradec Králové, Charles University, Akademika Heyrovského 1203/8, 500 05 Hradec Králové, Czech Republic*

E-mail: marie.skepo@compchem.lu.se

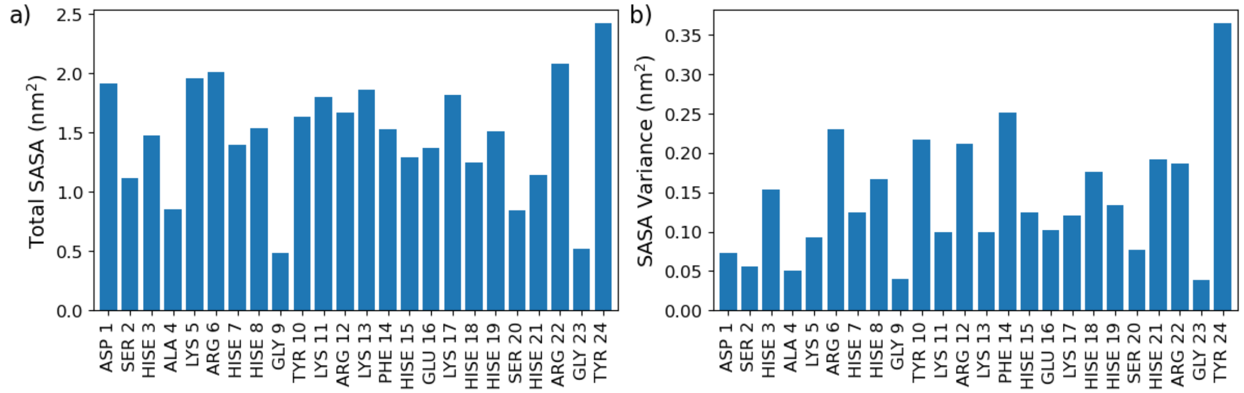

Figure S1: Bar plots of the respective solvent accessible surface area, SASA, depicted as (a) sums and (b) variance by residues.

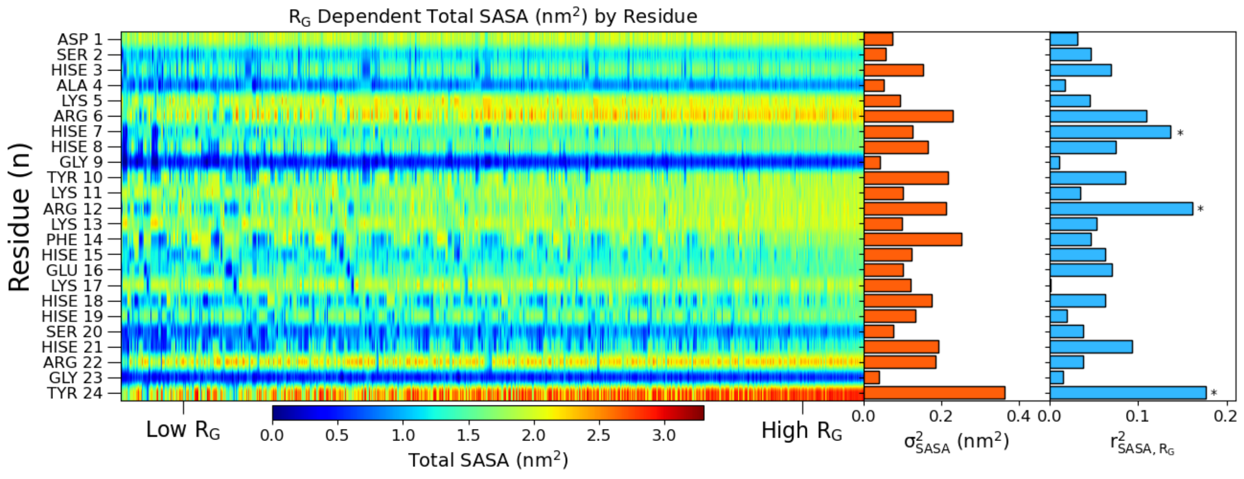

Figure S2: Heatmap of the total solvent accessible surface area, SASA, by residues as a function of increasing radius of gyration, R_g, the respective variance by residues, and the correlation between the residue's SASA and R_g.

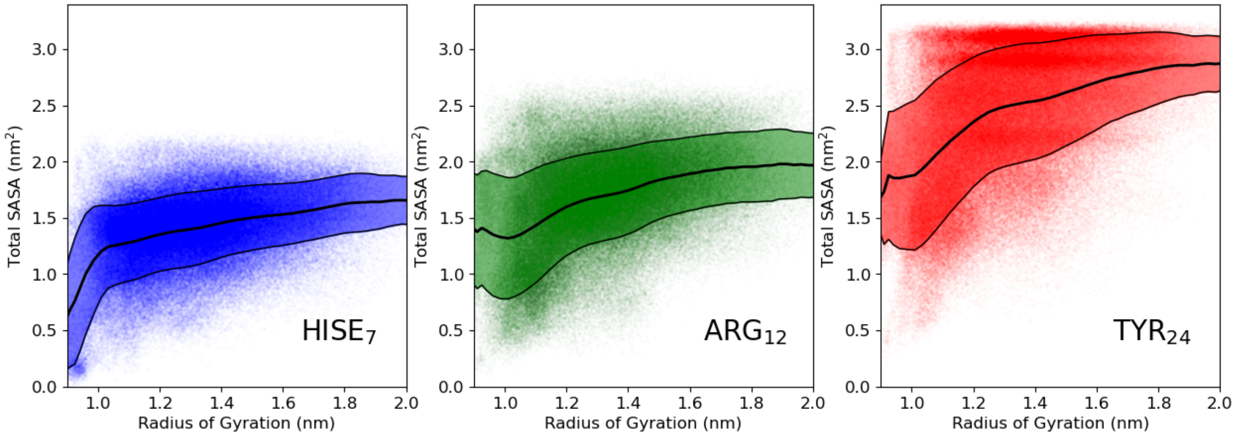

Figure S3: Residues which show an unusually strong correlation between the radius of gyration, R_g, and total solvent accessible surface area, SASA.

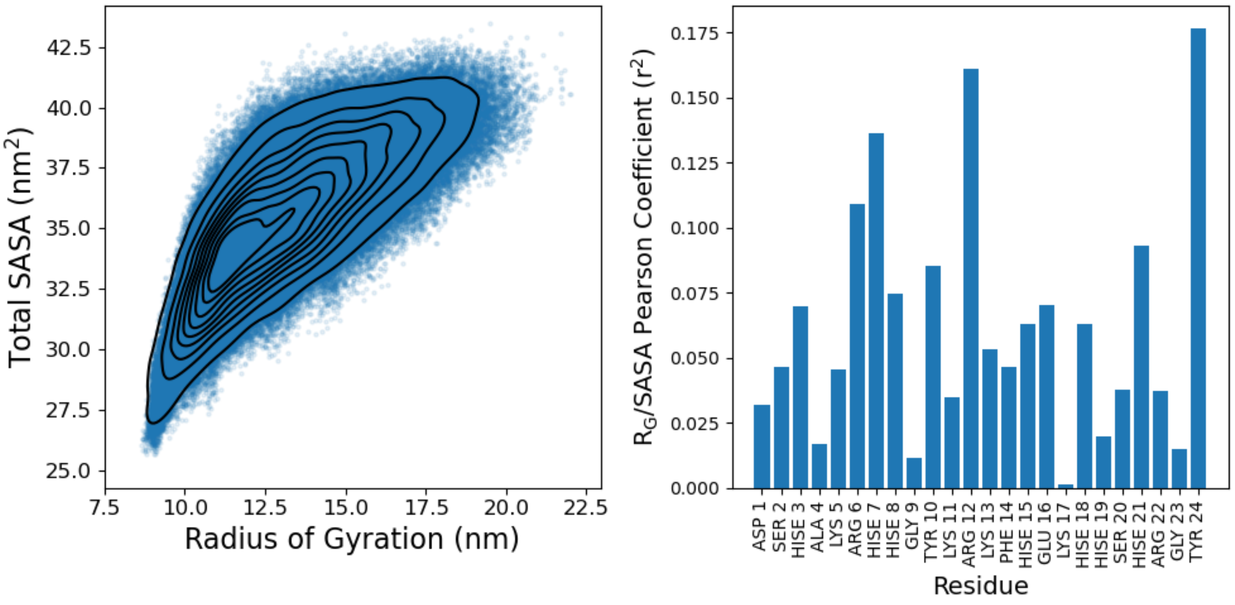

Figure S4: Plot of the radius of gyration, R_g, of the frames from the trajectory, and their corresponding total solvent-accessible surface area, SASA.

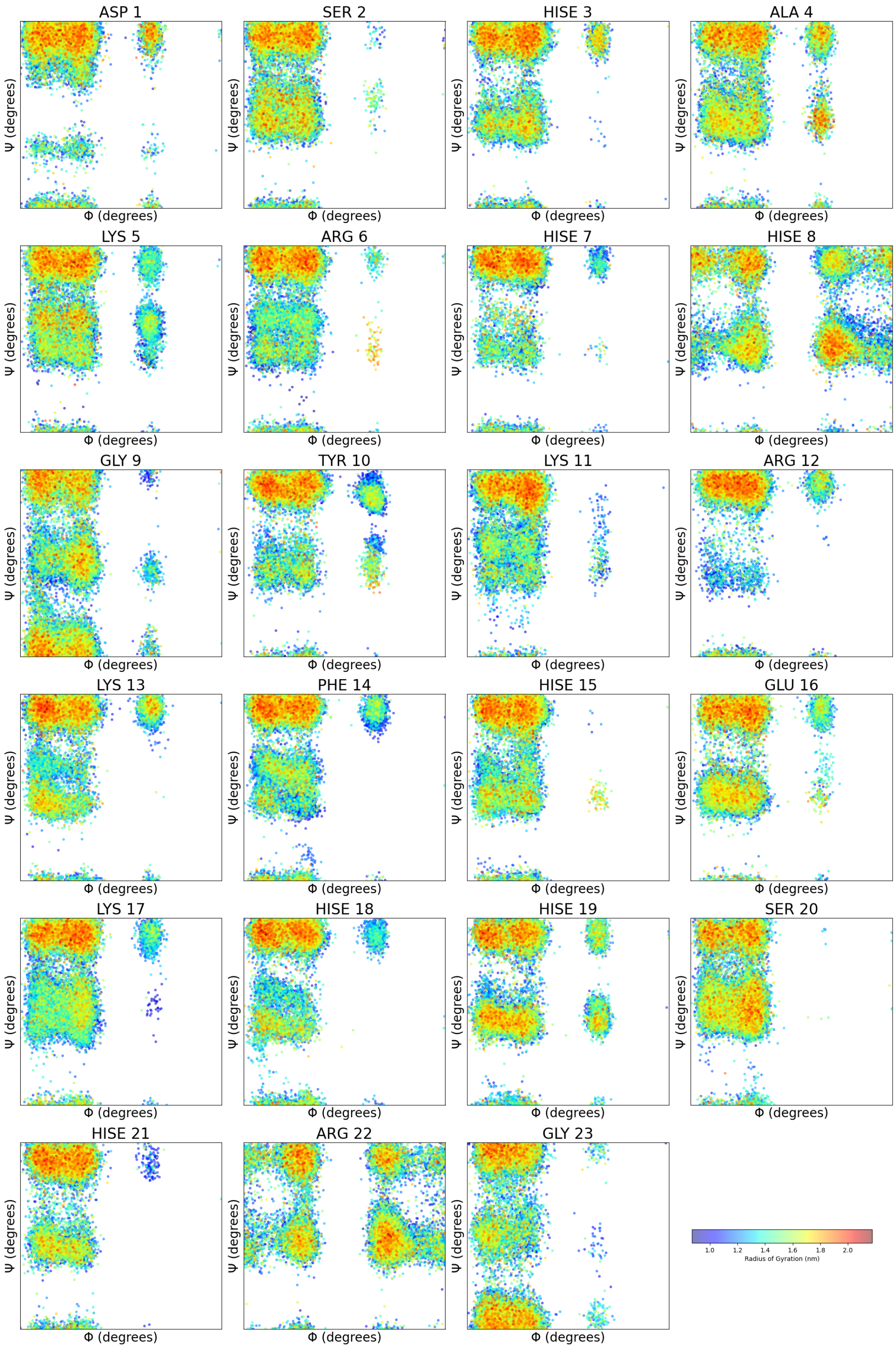

Figure S5: Ramachandran plots from each of the residues plotted according to their radius of gyration, R_G.

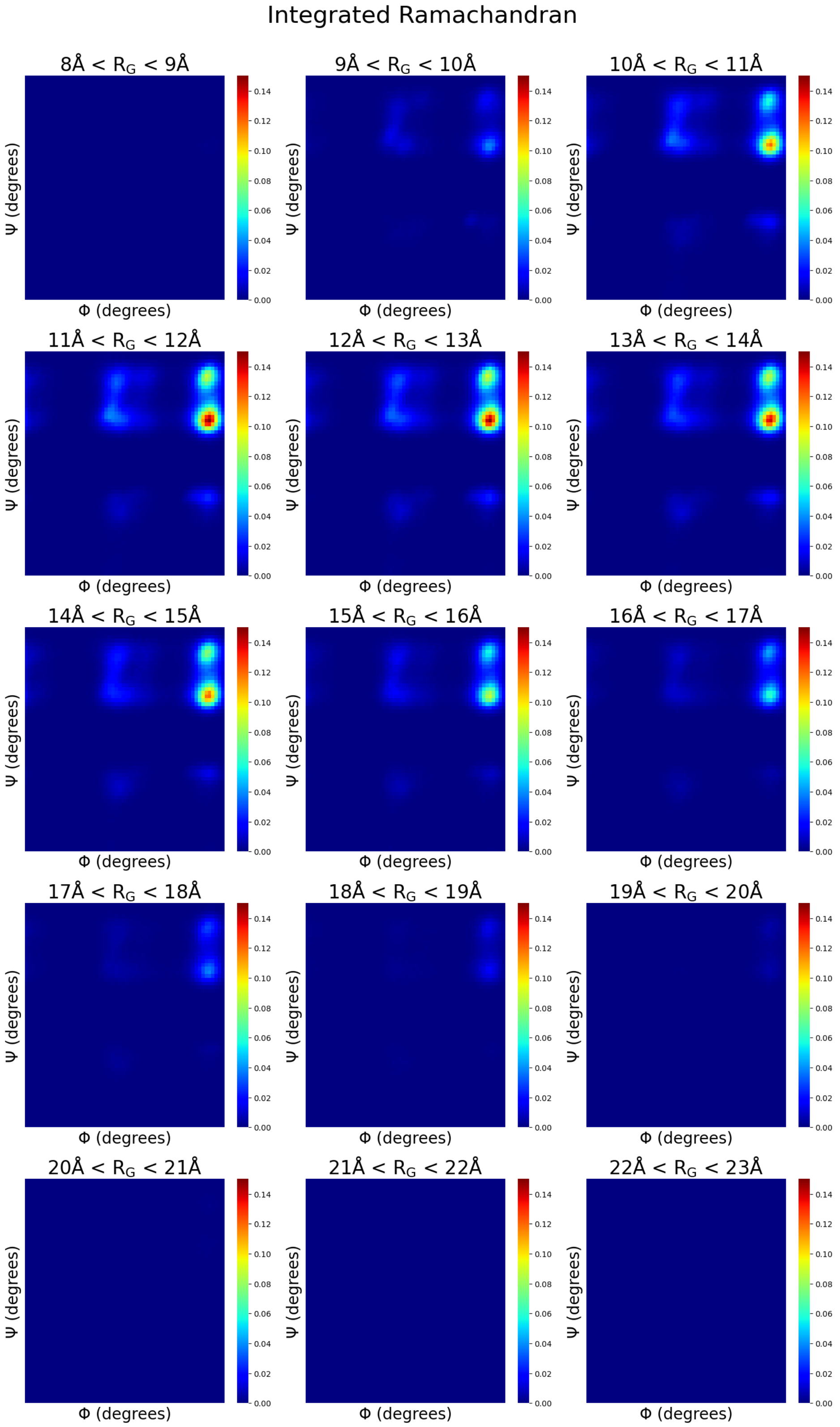

Figure S6: Ramachandran plots from each of the radius of gyration, R_g, groups as a heatmap distribution.

Table S1: Percentages of secondary structure elements determined via the DSSP algorithm.

R_g [Å]	PPII-helix [%]	Strand [%]	3-helix [%]	β-bridge [%]	α-helix [%]	H-bond turns [%]
8	1.44	0.40	0.33	0.46	0.00	26.4
9	3.05	1.00	1.52	0.62	0.43	18.1
10	3.65	0.93	0.83	0.70	0.33	13.7
11	3.97	0.58	0.24	0.63	0.14	12.0
12	4.00	0.54	0.21	0.73	0.08	10.9
13	4.32	0.46	0.08	0.49	0.09	10.0
14	4.98	0.48	0.01	0.41	0.07	8.96
15	5.74	0.45	0.00	0.33	0.10	7.59
16	6.43	0.30	0.00	0.23	0.06	5.63
17	6.96	0.17	0.00	0.20	0.02	4.21
18	7.68	0.09	0.00	0.08	0.00	2.41
19	7.68	0.01	0.00	0.00	0.00	1.22
20	7.44	0.00	0.00	0.00	0.00	0.78
21	6.66	0.00	0.00	0.00	0.00	0.00
22	5.50	0.00	0.00	0.00	0.00	0.00
